# Physiological responses of the monocled cobra (*Naja kaouthia* Lesson, 1831) including venom production, to high ambient temperature exposure

**DOI:** 10.1590/1678-9199-JVATITD-2024-0058

**Published:** 2025-02-14

**Authors:** Taksa Vasaruchapong, Narongsak Chaiyabutr, Thanida Nampimoon, Sumpun Thammacharoen

**Affiliations:** 1 Department of Physiology, Faculty of Veterinary Science, Chulalongkorn University, Bangkok, Thailand.; 2 Queen Saovabha Memorial Institute, The Thai Red Cross Society, Bangkok, Thailand.

**Keywords:** Corticosterone, High ambient temperature, Naja kaouthia, Phospholipase A_2_, Venom production.

## Abstract

**Background:**

Temperature regulation is essentially important for survival of poikilotherms such as snakes. Body temperature is regulated by snakes through behavioral and physiological responses. The global-warming crisis, combined with the need to house large population of snakes in limited spaces, increases the likelihood of exposing snakes to high ambient temperature (HTa), requiring it reliance on physiological responses. This study aimed to study the effect of HTa exposure on physiological responses and venom production, which have rarely been studied.

**Methods:**

Eleven adult monocled cobras (*Naja kaouthia* Lesson, 1831) were divided into two groups. The concurrent control group was housed in a temperature-controlled room, and the heat exposed group was housed in the same room with gradually increasing temperatures (25°C-35°C) for 4 h on four consecutive days. Data were collected 3 days before the experiment as the baseline and then compared with day 1 and day 4 after HTa exposure data representing immediate and prolonged effects. Body temperature, body weight, water intake, heart rate, hematology, plasma biochemistry, body-fluid compartments, hormonal response, heat shock protein expression and venom production were measured.

**Results:**

In response to HTa exposure, body temperature and heart rate increased, plasma volume significantly decreased, but water intake increased. Hematocrit and plasma protein progressively decreased in the latter stages of experimentation, but HTa diminished this effect. HTa only increased plasma corticosterone on day 1. Exposure to HTa increased venom protein concentration on day 4 and diminished the decreased proportion effect of frequent venom collection on phospholipase A_2_ component.

**Conclusion:**

Increased heart rate and fluid shift from the intravascular compartment appeared to be the underlying mechanism for heat dissipation during HTa exposure. Under the study condition, HTa caused heat stress, but the snake could adapt after continued exposure. Additionally, HTa increased venom protein concentration in *N. kaouthia*, particularly phospholipase A_2_ component.

## Background

Temperature regulation by snakes is important for their survival. Reptiles potentially regulate their body temperature by selecting their appropriate environmental temperature, which is known as behavioral thermoregulation [1]. Reptiles can also perform physiological adjustments in order to keep their body temperature stable under fluctuating environments [2]. In previous decades, snakes have been introduced to captive environments, such as for exotic pets or for medical research [3]. These research efforts require captive maintenance of relatively large numbers of snakes in limited spaces; this can diminish behavioral thermoregulation. Also, the global-warming crisis has increased the risk of heat accumulation, thereby exposing snakes to prolonged elevated temperatures and increasing their reliance on physiological responses as well as regulation. Several studies have investigated the high ambient temperature (HTa) effect on the physiological responses of reptiles. The results showed that HTa increased heart rate along with cutaneous vasodilation to augment heat dissipation [2] and raised plasma corticosterone (CORT) levels [4-5]. However, studies on the effect of HTa on venom production are very scarce, especially of medically important Southeast Asian species.

The monocled cobra (*Naja kaouthia* Lesson, 1831) is a medically important venomous snake native to Thailand and several Asian countries. Annually, *N. kaouthia* is the most common cause of neurotoxic envenomation in Thailand every year [6]. The *N. kaouthia* venom (NKV) contains many components, but 70% consists of 3-finger-fold toxins (3-FFTX) and phospholipase A_2_ (PLA_2_) [7]. The venom composition can vary due to several factors, such as preferred prey, age, sex, and geographic origin. Venom variability occurs on several levels: from individual specimens to family rank [8]. NKV variability has been widely investigated primarily to determine the cross-neutralizing capacities of available antivenom [7]. A few studies have demonstrated the effect of HTa on venom variability in which the summer season or under artificial heating could increase venom yield and protein concentration [9] but did not affect venom composition when studied in individual specimens [10]. In contrast, modern proteomic studies suggested that venom composition can vary with different captive temperatures and the time of venom replenishment [11]. Therefore, the ambient temperature effect on venom composition and its underlying mechanism are not known conclusively.

The present experiment aimed to study the physiological responses when exposed to immediate and prolonged HTa and its effect on venom production in *N. kaouthia*. The hypotheses of the experiment were, firstly, HTa alters the physiological responses to increase heat dissipation resulting in water loss and changes in plasma biochemistry which affect the body fluid compartments. Secondly, the water loss could decrease the venom yield and affect the venom composition.

## Methods

### Animal management

This study used 11 specimens of *N. kaouthia* consisting of eight males and three females from the snake farm of the Queen Saovabha Memorial Institute (QSMI). All snakes had a snout-to-vent length exceeding 1 m and a body weight of approximately 1 kg [12]. The snakes exhibited normal appetite and showed no clinical abnormalities upon physical examination. The snakes were fed once every 2 weeks with prefrozen frogs at approximately 10% of the snake's body weight and underwent a 1-week fasting period before the snakes were prepared. Water was offered in a ceramic bowl *ad libitum*. All snakes were housed in a temperature-controlled room from 8:00 a.m. to 4:00 p.m. with a typical temperature/humidity was 25°C ± 2°C/55% ± 5% without an additional heat source, after which they were exposed to the natural ambient conditions. The light/dark cycle was 12/12 hours. The ambient conditions were relatively stable throughout the year. Each snake was housed individually in a lockable, transparent acrylic enclosure measuring 60 × 45 × 30 cm (L × W × H). The enclosure featured ventilation slits measuring 40 × 30 cm on the upper wall and 40 × 15 cm on the left and right walls, secured by 0.5-cm plastic-coated wire mesh. The front and back walls featured ventilation holes with a diameter of 0.5 cm. A dark hiding box measuring 35 × 23.5 × 7 cm was provided. The experiment was conducted at the Snake Farm, QSMI, Thailand. The procedures used in this study were performed according to the guidelines approved by the Animal Care and Use Committee of QSMI (#09-2021).

### Animal preparation 

Before the experiment, all snakes underwent anesthetization using the open-drop technique [13] with isoflurane (Attane, Piramal Critical Care Inc., Bethlehem, Pennsylvania, USA) for the surgical placement of intrajugular catheters adapted from Vasaruchapong et al. [14]. One catheter was inserted toward the head direction (Cat-1) for blood collection. In the body-fluid compartment study, another catheter was inserted toward the heart direction (Cat-2) to inject markers and administer reconstituted blood, which are described in the next section. A thermosensitive microchip (Lifechip; Destron Fearing Corp., Minnesota, USA) for monitoring body temperature (Tb) was implanted in the body coelom on the left-lateral side, positioned 15 ventral scales cranial to the anal plate. Two bands of adhesive tape were placed on the ventral scales cranial and caudal to heart positions for tallying the heart rate. The snakes were monitored for three days after surgery. A single intramuscular injection of tramadol (Tramadol, T.P. Drug Laboratories (1969), Bangkok, Thailand) at a dosage of 5 mg/kg was administered for analgesia on postoperative day 1 [15]. Only snakes displaying normal behavior, without signs of decreased motor activity, motion stiffness, or clinically significant bleeding, were included in the experiment.

### Experimental design

**Figure 1.**
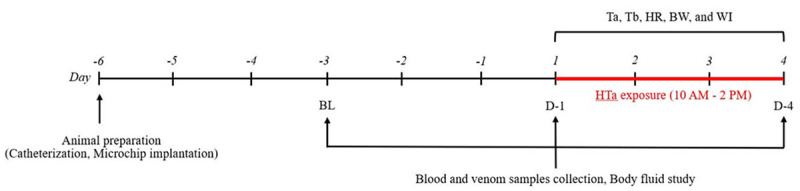
Schematic representation of the experimental timeline showing that the baseline data (BL) is composed of sample collection at 3 days before the experiment and was followed by collection at day 1 (D-1) and day 4 (D-4) after terminating high ambient temperature (HTa) exposure. The ambient temperature (Ta), body temperature (Tb), heart rate (HR), body weight (BW), and water intake (WI) were monitored during the experimental period.

The snakes were randomized into two groups: one concurrent control (CC) group consisting of six cobras and one heat exposed (HE) group consisting of five cobras. The CC group was housed in a temperature-controlled room maintained at 25°C ± 1°C from 8:00 a.m. to 4:00 p.m., after which they were exposed to natural ambient temperatures averaging 25.7°C ± 0.3°C. The HE group was housed in the same room and exposed to HTa in the heating chamber for four consecutive days. The heat-exposure pattern was from 10 a.m. to 2 p.m.. The temperature was gradually increased from 25°C to 35°C (ΔT = 10°C), after which the snakes were maintained at the same temperature as for the CC group. The blood samples, venom samples, and body-fluid study were performed 3 days before heat exposure as a baseline (BL) and at the end of the HTa exposure period of day 1 (D-1) and day 4 (D-4), to represent the immediate and prolonged effects of HTa exposure, respectively. The ambient temperature (Ta), and Tb were recorded from 9:00 a.m. to 5:00 p.m. for monitoring the hourly change. The heart rate could be observed only from 10:00 a.m. to 2:00 p.m. because of time limitation due to subsequent blood collection. Body weight and water intake were recorded every morning. A schematic representation of the experimental timeline is shown in Figure 1. 

*Heat source and temperature measurement*

A 150-watt ceramic radiant heater (Elstein Infrared Elements, Elstein-Werk, Northeim, Germany) controlled by a thermostat set at 35°C was placed above the enclosure as a heat source. A digital thermometer (Xiaomi MIjia Thermometer 2, Xiaomi Inc, Beijing, China) was placed inside the hiding box for monitoring Ta. The Tb was acquired by reading the thermosensitive microchip of a microchip reader (Destron Pocket-Reader; Destron Fearing Corp., South Saint Paul, Minnesota, USA) capable of reading the microchip from outside the enclosure.

*Determination of the body weight and water intake*

The snake was weighed in a plastic bucket, and the actual body weight was calculated by subtracting the weight of empty bucket from that with the snake. Two water bowls with the same dimensions were placed at the front part of the enclosure. One drinking bowl provide free access drinking, whereas another control bowl was covered by a plastic-coated wire mesh, preventing the snake from drinking but allowing for natural evaporation. Both water bowls were weighed every morning, and the water intake was calculated by subtracting the control bowl’s weight from the drinking bowl’s weight. 

*Heart rate*

The heart rate was recorded by tallying the number of heartbeats in 1 min by observing the movement of the ventral scales at the heart position. The observation was performed beneath the transparent enclosure without disturbing the snakes. 

*Blood sample collection*

Three milliliters of venous blood were collected via Cat-1 without handling the snakes to avoid interference of the handling effect on the CORT level. The blood collection was performed at the same time (2:00 p.m.) to avoid the daily fluctuation pattern of CORT. Blood samples were collected in a plastic test tube containing lithium heparin as an anticoagulant for further measurements. A 1.5 mL blood sample was used for hematology and heat shock protein (Hsp) determination. Another 1.5 mL sample was spun at 3,000 × g for 5 min to separate the plasma, kept at −20°C for further plasma biochemistry and hormone determination, and as a control for body-fluid compartmental analysis. The precipitated blood was reconstituted with Ringer’s solution (R-cetate, General Hospital Products Public Co., Ltd., Pathumthani, Thailand) added to an equal volume of 3 mL and administered to the snake via Cat-2 to replace the fluid loss.

*Venom collection and venom yield*

Venom collection was performed after blood collection. The Petri dish covered with stretched parafilm was placed against the lips to stimulate two continuous voluntary bites and venom injection. The venom yield was a venom weight in milligrams calculated by subtracting the empty Petri dish weight from the weight of the Petri dish with venom.

### Determination of the body-fluid 

The markers for body-fluid study determination experiment were injected via Cat-2 after venom collection. The plasma volume (PV), extracellular fluid (ECF) and total body water (TBW) were studied by use of Evans blue (Fluka Chemie GmbH, Buchs, Switzerland), sodium thiocyanate (NaSCN) [16] (Avantor, Dublin, Ireland) and urea [17] (Sigma-Aldrich Chemie GmbH, Steinheim, Germany) as markers. The intracellular fluid (ICF) was calculated by subtracting ECF from TBW. A single dose of 1 mL/kg of the marker solution consisting of 0.1% Evans blue, 7.5% NaSCN and 1.5% urea was injected. The actual dose administered was determined by subtracting the empty syringe’s weight from the weight of the syringe with the marker solution. 

Two milliliters of a blood sample for body-fluid determination were serially collected from Cat-1 at 30, 60, 90 and 120 min after marker injection. The blood sample was collected in a plastic test tube containing lithium heparin as an anticoagulant. After each collection, the blood was immediately spun to separate the plasma and kept at −20°C for further analysis. To substitute for blood loss, the precipitated blood cells were reconstituted with Ringer’s solution to an equal volume of 2 mL of whole blood and administered to the snake via Cat-2 before subsequent blood collection. Evans blue in the plasma was measured at a wavelength of 625 nm by a spectrophotometer. Plasma NaSCN and urea concentrations were determined by performing the methods of Medway and Kare [18] and Fawcett and Scoott [19], respectively. The concentration of each marker was plotted against the time course after marker injection on a semi-logarithmic scale, and the dilution of each marker was determined by extrapolation for the concentration at the theoretical zero time of complete mixing of the marker. The volume of fluid in each compartment was calculated by dividing the actual injected marker (mg) by the sample concentration (mg/mL) at zero time. 

### Determination of the blood and plasma parameters

*Hematocrit and plasma biochemistry*

The hematocrit (Hct) was obtained by filling a microhematocrit tube with blood and spinning at 12,000 × g for 5 min, then reading from the Hct reading chart. The uric acid, total plasma protein (TP), sodium (Na^+^), potassium (K^+^), and chloride (Cl^-^) were measured by an automated clinical chemistry analyzer (AU400 Olympus Biochemistry Analyzer, Beckman Coulter, Brea, California, USA).

*Plasma CORT*

Plasma CORT was measured by use of a sheep polyclonal antibody-based competitive enzyme-linked immunosorbent assay ELISA [14], following the manufacturer’s instructions (Corticosterone Multi-Format ELISA Kits; Arbor Assays, Ann Arbor, Michigan, USA). The detection limit of the kit was 7.7 pg/mL. The CORT concentration was measured and calculated by a microplate reader (Sunrise; TECAN, Männedorf, Switzerland) with built-in 4PLC data analysis software (Magellan; TECAN, Männedorf, Switzerland). The coefficient of variation of the dilution linearity of the pooled serum was 10.24%. The intraassay variability was 9.59%.

*Heat shock protein 70 (Hsp-70) gene expression*

Total RNA was extracted from whole blood use of Trizol reagent (TRIzol™ Reagent, Thermo Fisher Scientific Inc., Waltham, MA, USA). The RNA concentration was measured by use of an RNA quantification kit (Qubit RNA High Sensitivity (HS) Assay Kit, Thermo Fisher Scientific Inc., Waltham, Massachusetts, USA) and kept at −20 ºC for further cDNA synthesis. An RNA concentration of 300 ng was used for first-strand cDNA synthesis using a cDNA synthesis kit (RevertAid First Strand cDNA Synthesis Kit, Thermo Fisher Scientific Inc., Waltham, MA, USA). Quantitative SYBR Green real-time polymerase chain reaction (RT-PCR) was performed with Light Cycler (CFX96 Touch Real-Time PCR Detection System, Bio-Rad Laboratories Ltd., Hercules, California, USA). Hsp-70 expression was estimated by performing RT-PCR (SsoAdvanced Universal SYBR Green Supermix, Bio-Rad Laboratories Ltd., Hercules, California, USA). The primer sequence of Hsp-70 was obtained from another reptile species, the Chinese soft-shelled turtle [Pelodiscus sinensis (Wiegmann, 1835)], based on GenBank accession no. JN582024 [20]. The reaction was performed under the following conditions: 95°C for 30 s followed by 40 cycles of 95°C for 10 s, 55°C for 10 s, and finally 72°C for 30 s. The melt-curve protocol was followed by 10 s of 95°C and then 5 s each at 0.5°C increments between 65°C to 95°C. The brain-derived neurotrophic factor (BDNF) from the Chinese cobra (*Naja atra* Cantor, 1842) was considered a housekeeping gene for amplification under the same conditions. The BDNF sequence was based on GenBank accession no. KX694740 [21]. Evaluation of the gene expression levels relies on the comparative threshold cycle method referred to as the 2^−∆∆Ct^ method [22]. The result was presented as a fold change from the BL level within the group.

### Determination of the venom protein concentration and composition

The venom protein concentration was determined by performing Bradford protein assay (Pierce Bradford Protein Assay Kit, Thermo Fisher Scientific Inc., Waltham, MA, USA) with bovine serum albumin as a standard. A 30 µg amount of total protein from each sample was subjected to reversed-phase high-performance liquid chromatography (RP-HPLC) (Agilent 1100 series HPLC system, Agilent Technologies, Santa Clara, CA, USA) using a C-18 column (4.6 mm × 250 mm) (Agilent Zorbax 300SB-C18, Agilent Technologies, Santa Clara, CA, USA). The venom was eluted according to the HPLC method conditions of Lomonte and Calvete [23] The peak signals were analyzed by use of commercial computer software (Agilent ChemStation version A.09.01, Agilent Technologies, Santa Clara, CA, USA), and the data were presented as a percentage of the area under the curve (%Area) of each peak to the total area under the curve. The known major components of NKV (the neurotoxin (NTX) and phospholipase A_2_ (PLA_2_) fractions) acquired from QSMI were eluted according to the same HPLC methodology for comparison. 

### Statistical analyses

The commercial computer software GraphPad, Prism 8.0 (GraphPad Software, Boston, MA, USA) was used for data analysis and scientific graphing. The data of each sampling point were presented by means with the standard error of the mean. Pearson’s correlation model was used to determine the correlation coefficient to evaluate the correlation between Tb and HR. A linear mixed model followed by Tukey’s test for pairwise comparisons were used to compare the data of each sampling point with the BL. The normality of the distribution of the residuals from the model was assessed by performing the Shapiro-Wilk test. The comparison between the CC and HE groups at each sampling point used an unpaired Student’s *t*-test for separate analysis. 

## Results

### Ambient temperature and the effect of HTa on body temperature and heart rate 

**Figure 2.**
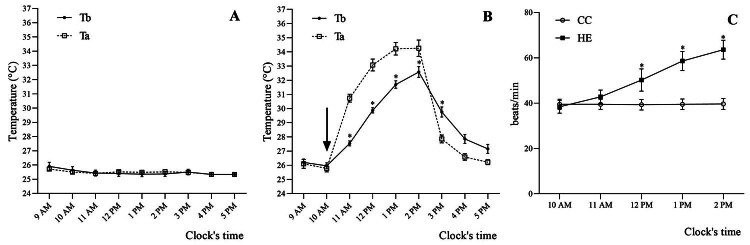
The ambient temperature (Ta) and body temperature (Tb) patterns of **(A)** the concurrent control (CC) group and **(B)** heat-exposed (HE) group are shown. The Tb of the CC group showed no significant difference from the level at the initial time of 10 a.m., whereas the Tb of the HE group were gradually increased after Ta. **(C)** The heart rate (HR) patterns of the CC and HE groups found significantly increase HR in the HE group from 12:00 a.m. to 2:00 p.m. *Different from the initial time (*p* < 0.05).

The four-day average Ta, Tb, and HR at the same clock time were used in the analysis. The data at each clock time was compared to the data at the initial time (10 a.m.). The average Ta at the initial time of the CC group was 25.5ºC ± 0.1ºC with no significant change during the experiment. The average Ta of the HE group increased from 25.8ºC ± 0.2ºC to the highest temperature of 34.2ºC ± 0.6 ºC at 2 p.m. (ΔT = 8.4°C). The Tb of the CC group was not significantly different from that at the initial time (F_7,35_ = 1.80, *p* > 0.05) (Figure 2 A), whereas the Tb of the HE group that was subjected to Ta change was significantly higher than the initial time Tb (10 a.m., 26.0ºC ± 0.2ºC) after 11 a.m. (27.5 ºC ± 0.2 ºC) (q_28_ = 14.51, *p* < 0.05) to 3 p.m. (29.7 ºC ± 0.3 ºC) (q_28_ = 6.08, *p* < 0.05) (Figure 2 B). The heart rate showed the same response to Ta, which was stable in the CC group, but gradually increased significantly above the initial rate in the HE group (10 a.m., 38 ± 3 times/min) after 12 p.m. (43 ± 3 times/min, q_16_ = 7.15, *p* < 0.05) to 2 p.m. (63 ±4 times/min, q_16_ = 15.26, *p* < 0.05) (Figure 2 C). The Tb of the two groups were found to be strongly and positively correlated with heart rate (*r* = 0.703, *p* < 0.05).

### Effect of HTa on body weight, water intake, body-fluid compartments

**Figure 3.**
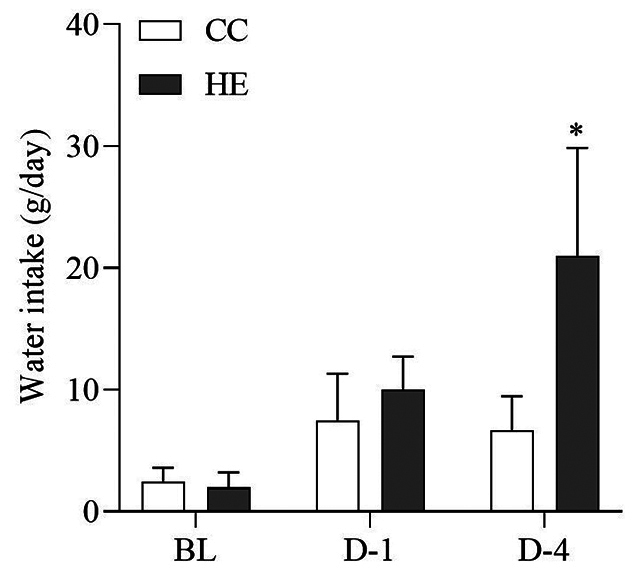
Water intake at baseline (BL), day 1 (D-1) and day 4 (D-4) of the concurrent control (CC) group and heat-exposed (HE) group. The HE group had significantly increased water intake on D-4 relative to BL. *Different from baseline (*p* < 0.05).

**Figure 4.**
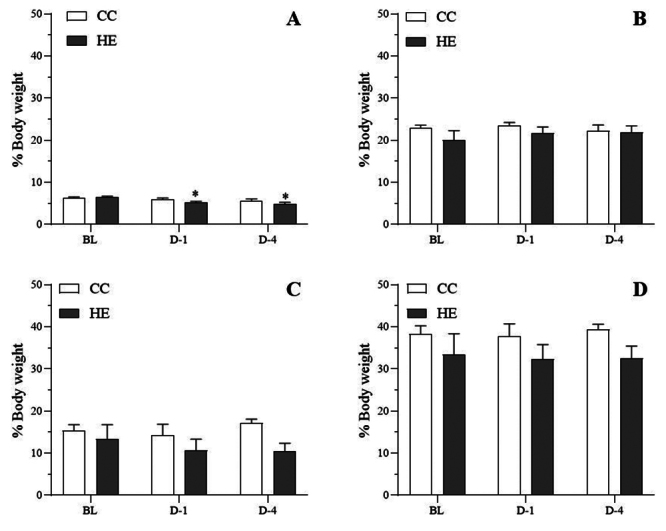
The body fluid in each compartment of the concurrent control (CC) group and heat-exposed (HE) group. **(A)** The HE group showed a significant decrease in the plasma volume on day 1 (D-1) and day 4 (D-4) relative to the baseline level (BL). **(B)** The extracellular fluid, **(C)** intracellular fluid, and **(D)** total body water was the same at each time point. *Different from baseline (*p* < 0.05).

The average body weights of the CC and HE groups at BL were 0.94 ± 0.08 kg and 1.09 ± 0.08 kg, respectively, with no significant change at all sampling points (F_2,18_ = 0.29, *p* > 0.05). The water intake was significantly increased by the effect of the day of the experiment (F_2,18_ = 4.45, *p* < 0.05). The CC group showed no significant change in water intake at all sampling points (q_18_ = 1.35 and 1.12, *p* > 0.05) but the HE group showed a significant increase in water intake on D-4 (q_18_ = 4.67, *p* < 0.05) (Figure 3). Regarding the different body weights of each snake, the volume of body fluid in milliliters of each compartment was calculated as percentages of the body weight and used for analysis. The effect of the day of the experiment in both groups tended to decrease PV (F_2,18_ = 8.61, *p* < 0.05). The CC group showed no significant change in PV at all sampling points (q_18_ = 1.28 and 2.73, *p* > 0.05) but the HE group showed significantly decreased PV on D-1 and D-4 (q_18_ = 3.99 and 5.33, respectively, *p* < 0.05) (Figure 4 A). The ECF, ICF, and TBW were not significantly changed in either group (F_2,18_ = 1.27, 0.90 and 0.16, respectively, *p* > 0.05) (Figures 4 B to 4D).

### Effect of HTa on hematocrit and plasma biochemistry

Hct was significantly affected by the day of the experiment and HTa exposure, with a two-factor interaction effect (F_2,18_ = 15.60, 5.50 and 6.61, respectively, *p* < 0.05). The CC group showed a significant decrease in Hct on D-1 and D-4 (q_18_ = 5.22 and 9.06, respectively, *p* < 0.05), whereas the HE group showed no significant difference in Hct at all sampling points (q_18_ = 1.26 and 2.24, respectively, *p* > 0.05), and a separate analysis found that the HE group had significantly higher Hct than the CC group on D-1 (t_9_ = 4.84, *p* < 0.05). 

Plasma biochemistry showed different effects of several parameters between the CC and HE groups. The day of the experiment factor significantly increased uric acid, Na^+^, and Cl^−^ (F_2,18_ = 24.05, 5.14 and 8.50, respectively, *p* < 0.05), with uric acid also showing an interaction effect between the day of the experiment and HTa (F_2,18_ = 5.11, *p* < 0.05). On the contrary, the day of the experiment was associated with a significant decrease in TP (F_2,18_ = 4.45, respectively, *p* < 0.05) with an interaction effect between the day of the experiment and HTa (F_2,18_ = 7.05, *p* < 0.05). A pairwise comparison showed increases in uric acid on D-1, Na^+^ on D-4, and Cl on D-1 and D-4 in the CC group (q_18_ = 4.32, 3.95, 3.67 and 5.46, respectively, *p* < 0.05), but a significant decrease in TP on D-1 and D-4 (q_18_ = 0.58, and 2.30, respectively, *p* < 0.05). However, the pairwise comparison of the HE group showed only a significant increase in uric acid on D-1 and D-4 (q_18_ = 8.71 and 7.76, respectively, *p* < 0.05), whereas other parameters were unchanged. Only K^+^ remained unchanged at all sampling points (F_2,18_ = 0.29, *p* > 0.05). The hematology and plasma biochemistry data are presented in Table 1. 

**Table 1.** The hematology, plasma biochemistry and heat shock protein 70 (Hsp-70) expression. The hematocrit (Hct), uric acid, total plasma protein (TP), sodium (Na^+^), potassium (K^+^), chloride (Cl^−^) and heat shock protein 70 (Hsp-70) expression of the concurrent control (CC) group and heat-exposed (HE) group at baseline (BL), 1 day (D-1) after exposed to high ambient temperature (HTa) and 4 days (D-4) after HTa exposure.

	CC			HE			SEM	*P* **value**		
	BL	D-1	D-4	BL	D-1	D-4		HTa	Day	HTa x Day
Hct (%)	23.7^a^	19.0^b^	15.5^b^	22.6	23.9*	20.4	2.20	0.00	0.04	0.01
Uric acid (mg/dL)	2.4^a^	3.7^b^	3.1	1.7^c^	4.4^d^	4.1^d^	0.7	0.55	0.00	0.02
TP (g/dL)	6.1^a^	5.3^b^	4.9^b^	5.7	5.8	5.4	0.3	0.58	0.00	0.01
Na^+^ (mmol/L)	165.5^a^	171.2	172.5^b^	167.0	170.8	171.0	4.3	0.97	0.02	0.72
K^+^ (mmol/L)	5.1	5.3	5.8	5.6	5.3	5.7	0.9	0.77	0.43	0.75
Cl^−^ (mmol/L)	132.0^a^	138.8^b^	142.2^b^	134.4	137.4	140.2	4.6	0.93	0.03	0.49
Hsp-70 (Fold changed)	1.00	1.62	0.97	1.00	1.55	5.04	2.77	0.21	0.25	0.16

*p* value from linear mixed model analysis of variance and superscript letters showed the pairwise comparison using Tukey’s test, *p* < 0.05. *Different from the CC group at the same time point using unpaired Student’s *t*-test, *p* < 0.05.

### Effect of HTa on Hsp-70 expression and CORT 

**Figure 5.**
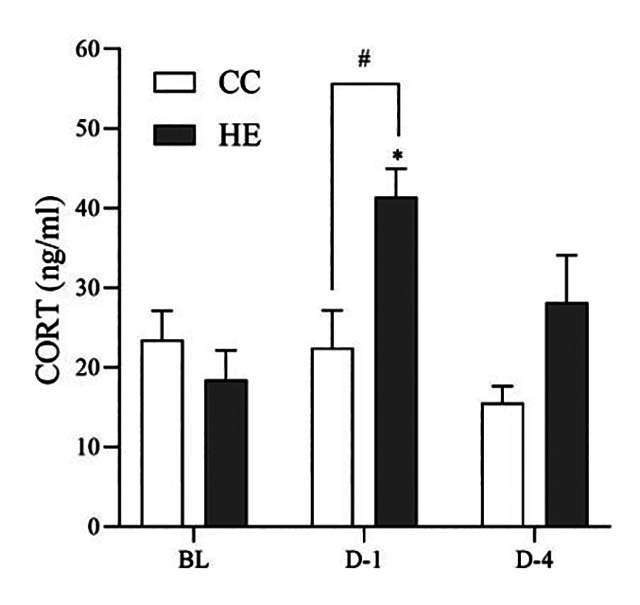
The effect of high ambient temperature on the plasma corticosterone (CORT) level of the concurrent control (CC) and heat exposed (HE) group. The HE group had significantly increased CORT on day 1 (D-1) of heat exposure relative to the baseline (BL) and significantly higher CORT than in the CC group, but there was no significant difference at day 4 (D-4). *Different from baseline (*p* < 0.05), ^#^unpaired Student’s *t*-test (*p* < 0.05).

There were no significant differences in Hsp-70 expression at any sampling points in The CC group and HE groups (Table 1; F_2,18_ = 2.00, *p >* 0.05). The CC group showed no significant changes in the CORT level at all sampling points (q_18_ = 0.24 and 1.88, respectively, *p* > 0.05), whereas the HE group had significantly increased CORT on D-1 (41.4 ± 3.6 ng/mL) relative to the BL value (18.5 ± 3.7 ng/mL) (q_18_ = 4.94, *p* < 0.05). This increased CORT level was also significantly higher on D-1 in the HE group than in the CC group in a separate analysis (t_9_ = 3.11, *p* < 0.05) (Figure 5). 

### Effect of HTa on venom yield and venom protein concentration

**Figure 6.**
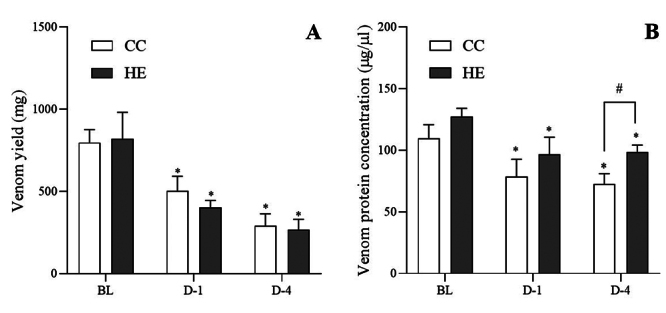
(A) The venom yield and (B) venom protein concentration for both the concurrent control (CC) and heat-exposed (HE) groups on day 1 (D-1) and day 4 (D-4) showed a progressive decrease from baseline (BL).

Venom yield and venom protein concentration were progressively and significantly decreased with increased days of the experiment (F_2,18_ = 46.81 and 13.97, respectively, *p* < 0.05). The venom yield of the CC group decreased from 796.67 ± 78.85 mg to 501.67 ± 89.76 mg on D-1 and 290.00 ± 73.30 mg on D-4 (q_18_ = 5.54 and 9.51, respectively, *p* < 0.05). The venom yield in the HE group decreased from 820 ± 161.18 mg to 402.00 ± 41.88 mg on D-1 and 266.00 ± 63.77 mg on D-4 (q_18_ = 7.16 and 9.49, respectively, *p* < 0.05) with no difference in venom yield between groups at each sampling point (t_9_ = 0.94 and 0.24, respectively, *p* > 0.05) (Figure 6 A). The venom protein concentration in the CC group decreased from 109.65 ± 11.07 µg/µL to 78.47 ± 14.27 µg/µL on D-1 and 72.54 ± 8.53 µg/µL on D-4 (q_18_ = 4.68 and 5.56, respectively, *p* < 0.05). The venom protein concentration in the HE group decreased from 127.47 ± 6.64 µg/µL to 96.60 ± 13.98 µg/µL on D-1 and 98.70 ± 5.41 µg/µL on D-4 (q_18_ = 4.22 and 3.94, respectively, *p* < 0.05). The separate analysis of venom protein concentration found that the HE group had a significantly higher protein concentration than the CC group on D-4 (t_9_ = 2.46, *p* < 0.05) (Figure 6 B).

### Effect of HTa on venom composition 

**Figure 7.**
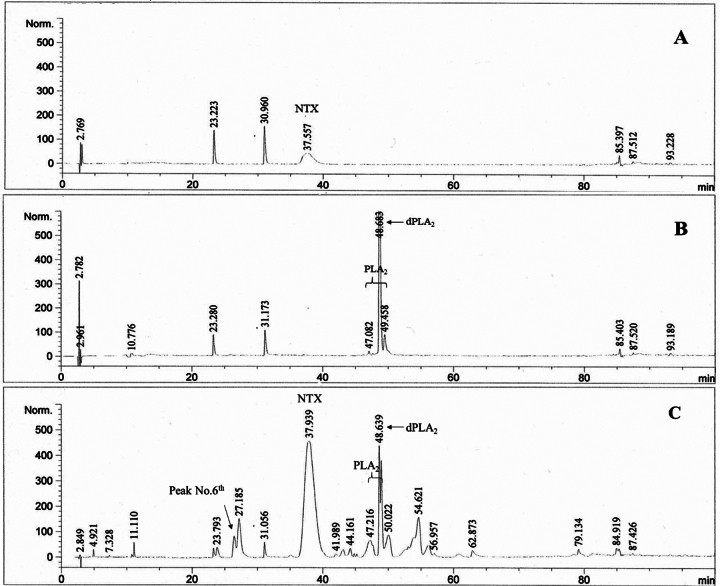
The chromatograms of **(A)** the known standard neurotoxin (NTX) and **(B)** phospholipase A_2_ (PLA_2_) components derived from *N. kaouthia*. The different isoforms of PLA_2_ were found in several connected peaks, with the most dominant peak (dPLA_2_) found at a retention time of approximately 48.6 min. The example of the baseline chromatogram of *N. kaouthia* in this study showed that **(C)** NTX was the most abundant, with PLA_2_ as the second most abundant component.

**Figure 8.**
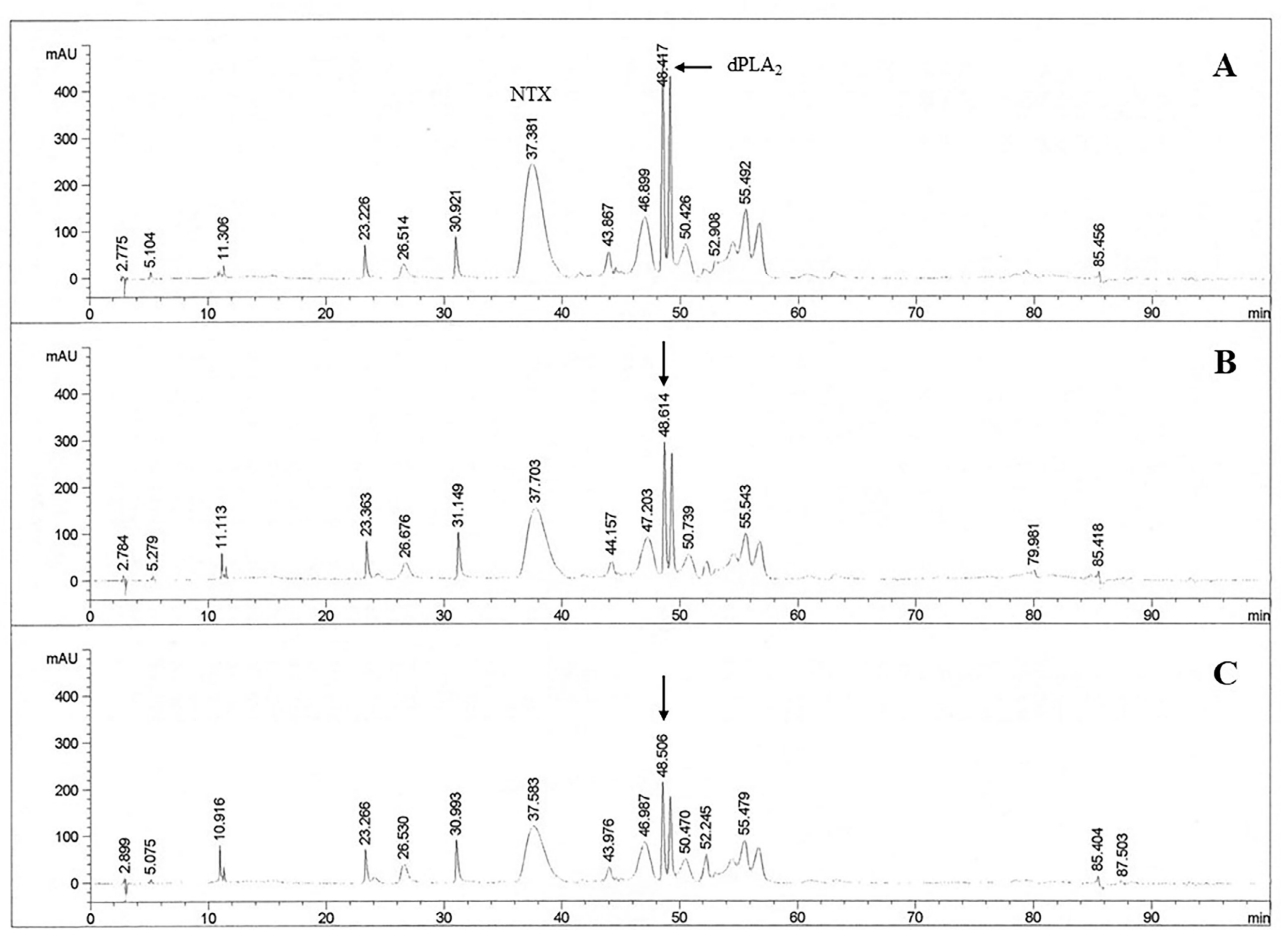
The chromatogram of *N. kaouthia* venom in the concurrent control (CC) group showing two major components: the neurotoxin (NTX) and the dominant phospholipase A_2_ (dPLA_2_) components. The proportion of the dPLA_2_ component (arrows), in comparison with **(A)** baseline, decreased on **(B)** day 1 and **(C)** day 4.

**Figure 9.**
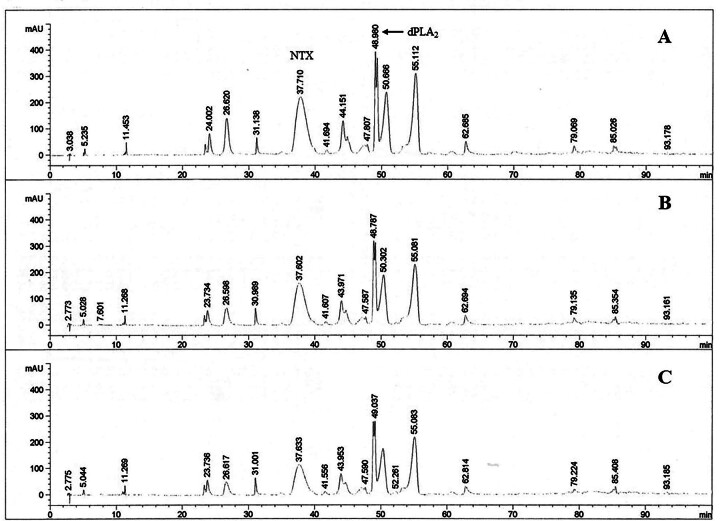
The chromatograms of *N. kaouthia* venom in the heat-exposed (HE) group on **(A)** baseline, **(B)** day 1 and **(C)** day 4. The proportions of the neurotoxin (NTX) and the dominant phospholipase A_2_ (dPLA_2_) components were the same at all time points.

**Figure 10.**
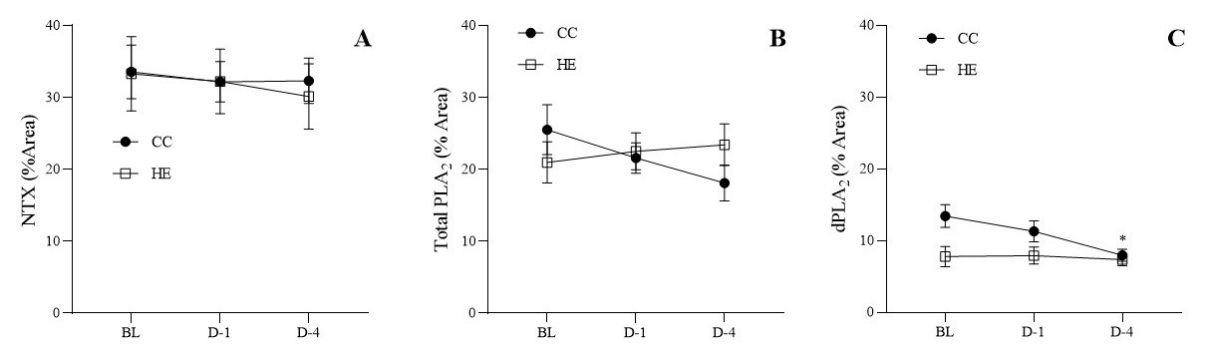
The proportion of major components in the *N. kaouthia* venom of the concurrent control (CC) group and heat-exposed (HE) group. **(A)** The neurotoxin (NTX) component levels were the same on day 1 (D-1) and day 4 (D-4) relative to baseline (BL). **(B)** The total phospholipase A_2_ (PLA_2_) component showed the opposite tendency between the CC group and HE group, of which the CC group showed a significant decrease in **(C)** the dominant phospholipase A_2_ (dPLA_2_) proportion on D-4. *Different from baseline (*p* < 0.05).

The chromatogram peaks were sorted by their average retention time and labeled with a peak number. The percent area under the curve (%Area) of each peak was averaged and used for further venom composition analysis. The HPLC chromatograms showed individual variations in venom composition among the subjects in the CC and HE groups. The CC group showed a total of 28 peaks, and the HE group showed a total of 31 peaks in the BL chromatogram. The known sample of NTX matched peak No.10 in the BL chromatogram at a retention time of 37.55 min (Figure 7 A). The known PLA_2_ fraction was detected as several connected peaks, which matched peak No.16-19 at retention times from 47.08-49.46 min, and the dominant peak (dPLA_2_) was No.18 at a retention time of 48.68 min (Figure 7 B). On the BL chromatogram, the NTX was the most abundant component in both the CC and HE groups which were 33.56% ± 3.73% and 33.29% ± 5.16%, respectively. The total PLA_2_ (peak Nos.16-19) was the second highest component of the CC and HE groups (25.52% ± 8.50% and 20.97% ± 2.87%, respectively), whereas the dPLA_2_ was significantly higher in CC group (13.43% ± 1.59%) than in the HE group (7.80% ± 1.40%) (t_9_ = 2.60, *p* < 0.05) (Figure 7 C). After HTa exposure, the proportion of dPLA_2_ progressively decreased in the chromatogram of the CC group on D-1 and D-4 (Figure 8), but this effect was not observed in the HE group (Figure 9). The %Area analysis showed no significant difference in NTX between the two groups at all sampling points (F_2,18_ = 0.82, *p* > 0.05) (Figure 10 A). However, total PLA_2_ tended to decrease in the CC group but tended to increase in the HE group (Figure 10 B). The dPLA_2_ was significantly affected by the day of the experiment (F_2,18_ = 6.37, *p* < 0.05), with an interaction between the day of the experiment and HTa effects (F_2,18_ = 4.54, *p* < 0.05). The dPLA_2_ in the CC group was significantly decreased to 7.97% ± 0.84% on D-4 (q_18_ = 4.18, *p* < 0.05), but this effect was not observed in the HE group (Figure 10 C). The CC group also showed a significant increase in the unidentified component, which was a peak No.6 at a retention time of 26.61 ± 0.17 min on D-4 (BL = 3.42% ± 0.96%, D-4 = 7.29% ± 2.13%) (q_18_ = 4.55, *p* < 0.05), but this change was not observed in the HE group. 

## Discussion

This study demonstrated that immediately and gradually increasing HTa (ΔTa = 8.4°C within 4 h.) caused significant effects on both physiological and behavioral responses and on venom production in *N. kaouthia*. Exposure to immediate or prolonged HTa significantly affected body temperature, heart rate, water intake, plasma volume, hematocrit, and total plasma protein, but only immediate HTa exposure could elevate the CORT level. HTa exposure also affected venom production. 

In mammals, the early responses to HTa are to increase heat dissipation and reduce heat production through behavioral and physiological adaptive responses before activating the hypothalamic-pituitary-adrenal (HPA) axis [24-26]. Heat stress refers to the combination of behavioral and physiological responses combined with HPA-axis activation [27-28]. The physiological responses of reptiles to HTa resemble those of mammals, with the aim of regulation body temperature [2]. The present study demonstrated that gradually increasing HTa exposure can cause heat stress in *N. kaouthia* by affecting the plasma CORT level. Although prolonged HTa exposure had a behavioral effect similar to that of immediate exposure, the CORT level was not different from the BL level. This might be an adaptation to HTa after continued exposure to HTa at some levels.

We observed that HTa increased Tb. The strong positive correlation between Tb and heart rate in the present study indicated the important role of blood circulation in heat dissipation as found in previous studies [2, 29]. The sympathetic nervous system was probably involved in the early physiological response by increasing the heart rate and dilating the peripheral blood vessels. Exposure to HTa also affected drinking behavior, which is a behavioral response also observed in mammals [30]. However, there are no reports of drinking behavior under heat stress conditions in reptiles, which would make these the first reported results of the HTa effect on the drinking behavior of *N. kaouthia*. 

Exposure to HTa significantly decreased PV, which indicated a fluid shift. However, the fluid in other compartments and body weight remained unaffected, and increased water intake might compensate for the water loss. The decreased PV response to HTa exposure suggests that evaporative cooling was an important underlying mechanism. The two major sites known for reptile evaporative water loss were cutaneous and respiratory water loss. The skin is the major site of water efflux in most terrestrial reptiles, but it appears to depend on physical conditions, such as skin permeability and ambient water vapor pressure [31, 32]. Respiratory water loss is substantial in lizards and monitors through gaping, but there is no evidence of a thermoregulation role in snakes, which have a very low respiratory rate [33]. However, we did not monitor the water loss from both sites in the present study; therefore, the mechanism of decreasing PV was inconclusive and requires further study. The TBW in this study was approximately 33%-38% of body weight at BL, which is lower than the 60%-75% of body weight previously reported in other reptiles [32]. Most studies have used the tritiated water dilution technique to measure TBW, but this study used urea instead because the tritiated water, which is radioactive, is now strictly regulated. Urea has been previously used in other snake species, such as the Reticulated Python (*Maloyopython reticulatus*) [34]. Urea has been widely used for studying TBW in mammals and has different distribution kinetics; thus, it is not directly comparable with tritiated water results [35]. Compared with mammals, snakes lack the same complement of urea-cycle enzymes and have only a small urea plasma level [32], which may account for their comparatively different urea metabolisms. Therefore, using urea as a marker for the TBW study in this experiment might explain the lower TBW than found in other methods, so it should be considered for use as a marker in investigations of reptiles. The use of urea to measure TBW in this study was unsuitable, so further investigations using alternative methods for measuring TBW are needed. Taken together, the decreased PV and increased water intake supported our hypothesis that HTa can affect body-fluid compartments. Additionally, the heart rate and fluid shift after HTa exposure suggests that evaporative cooling was an important underlying mechanism for heat dissipation in *N. kaouthia*.

The increased uric acid in the CC and HE groups was probably explained by the metabolism of the nitrogen-waste product in reptiles. Reptiles excrete nitrogen-waste products as a higher percentage of uric acid, which is less soluble in water and provides benefits as it conserves water [36]. The administration of urea as a marker in body-fluid experiment in our study possibly contributed to the uric acid increase in plasma. Urea was probably metabolized to uric acid, which is highly efficiently excreted by the kidneys of reptiles [37]. Thus, urea was unsuitable for measuring TBW in this study, and additional TBW investigations using other methods that do not affect nitrogen-waste products metabolism should be performed. The levels of Hct, TP, Na^+^ and Cl^−^ showed different tendencies between the groups. In the CC group, Hct and TP were significantly decreased but Na^+^ and Cl^-^ were significantly increased. The decrease in Hct could be the result of serial blood collection that accounted for 2% of the total blood volume at each sampling point. The decrease in TP might have been caused by the dilution effect of the repeated administration of reconstituted blood with Ringer’s solution that contained no nitrogen source; e.g., amino acids and proteins. The increase in Na^+^ and Cl^−^ might be related to the experimental protocol but could not be explained, so additional investigation is needed. The HE group showed stable levels of Hct, TP, Na^+^ and Cl^−^. The stable Hct in the HE group probably was because HTa can increase the number of red blood cells in circulation and diminish the effect in the CC group. The sympathetic alteration during HTa exposure might be the underlying cause of this effect. In mammals, epinephrine is known to increase Hct [38] by causing splenic contracture that acts as a red-blood cell reservoir [39]. The effect of epinephrine on Hct in ectothermy in the American bullfrog (*Rana catesbeiana*) also showed result equivalent to that in mammals [40]. The effect of epinephrine on Hct in reptiles probably involves increasing the relative red-blood cell volume for providing oxygen consumption [41] or other mechanisms that need further investigation. The stable TP level in the HE group would translate in the same manner for Hct in which HTa could increase TP. The increased TP might be a consequence of the fluid shift from the PV compartment, and TP was more concentrated, which was still inconclusive. These findings suggest that HTa could increase the Hct and TP levels in *N. kaouthia*.

The CC and HE groups showed a progressive decline in both venom yield and venom protein concentration, which was a consequence of the frequent venom collection protocol rather than the HTa effect. Frequent venom collection reportedly decreases the venom dry weight [9], which suggested that the snakes needed a longer time to substantially replenish their stored venom. Additionally, frequent venom collection in the puff adder (*Bitis arietans*) was previously found to affect the venom protein concentration [42]. Interestingly, the venom protein concentration was higher in the HE group than in the CC group on D-4. This higher protein concentration also coincides with the result of the venom composition study. The total PLA_2_ component tended to increase in the HE group with unchanged dPLA_2_. However, the total PLA_2_ component tended to decrease in the CC group and was accompanied by a significant decrease dPLA_2_. This effect might be explained by the asynchrony pattern of venom synthesis. Several studies have demonstrated that the time of venom replenishment and ambient temperature can affect the venom composition or the synthesis activity of each component in the venom [11, 42-44]. This is similar to the results of a study of venom from another elapid, the many-banded krait (*Bungarus multicinctus*), in which temperature affected the peak activity of PLA_2_ (β-bungarotoxin) replenishment [11]. Thus, the higher venom protein concentration and the tendency to increase total PLA_2_ in the HE group might lead to faster venom protein replenishment, particularly the PLA_2_ component. The sympathetic activation from the adaptive response to HTa might be the underlying mechanism of faster protein replenishment. The sympathetic nervous system reportedly was associated with venom production by triggering venom synthesis after venom ejection by activated α and β adrenoceptors [45-47]. Our study results showed that HTa exposure was significantly associated with increased venom protein concentration, and that PLA_2_ might be the main component that contributes to this finding.

## Conclusion

HTa exposure caused significant effects on the physiological response and venom production in *N. kaouthia*. HTa caused acute heat stress in *N. kaouthia*, but the snake was able to adapt to HTa after 4 days of continuous exposure. Frequent venom collection can reduce venom production and HTa exposure can diminish the decreased proportion effect of venom protein concentration, particularly of the PLA_2_ component. 

### Abbreviations

BL: baseline data; BDNF: brain-derived neurotrophic factor; Cat-1: intravenous catheter inserted toward head direction; Cat-2: intravenous catheter inserted toward heart direction; CC: concurrent control; Cl^-^: chloride; CORT: corticosterone; D-1: one day after heat exposure; D-4: four days after heat exposure; dPLA_2_: dominant phospholipase A_2_; ECF: extracellular fluid; Hct: hematocrit; HE: heat exposed; HPA: hypothalamic-pituitary-adrenal; Hsp-70: heat shock protein 70; HTa: high ambient temperature; ICF: intracellular fluid; K^+^: potassium; PLA_2_: phospholipase A_2_; Na^+^: sodium; NaSCN: sodium thiocyanate; NKV: *Naja kaouthia* venom; NTX; neurotoxin; PV: plasma volume; QSMI: Queen Saovabha Memorial Institute; Ta: ambient temperature; Tb: body temperature; TBW: total body water; TP: total plasma protein.
